# Simultaneous allergic traits in dogs and their owners are associated with living environment, lifestyle and microbial exposures

**DOI:** 10.1038/s41598-020-79055-x

**Published:** 2020-12-15

**Authors:** Jenni Lehtimäki, Hanna Sinkko, Anna Hielm-Björkman, Tiina Laatikainen, Lasse Ruokolainen, Hannes Lohi

**Affiliations:** 1 Research Programs Unit, Molecular Neurology, University of Helsinki, 00014 Helsinki, Finland; 2 Environmental Policy Centre, Finnish Environment Institute, 00790 Helsinki, Finland; 3 DogRisk and Helsinki One Health Research Groups, Department of Equine and Small Animal Medicine, Faculty of Veterinary Medicine, University of Helsinki, 00014 Helsinki, Finland; 4 National Institute for Health and Welfare, 00029 Helsinki, Finland; 5 Institute of Public Health and Clinical Nutrition, University of Eastern Finland, 70211 Kuopio, Finland; 6 Joint Municipal Authority for Social and Health Care in North Karelia, 80210 Joensuu, Finland; 7 Faculty of Biological and Environmental Sciences, University of Helsinki, 00014 Helsinki, Finland; 8 Department of Medical and Clinical Genetics, and Department of Veterinary Biosciences, University of Helsinki, 00014 Helsinki, Finland; 9 Folkhälsan Research Center, 00290 Helsinki, Finland

**Keywords:** Microbial communities, Allergy, Atopic dermatitis, Environmental impact

## Abstract

Both humans and pet dogs are more prone to develop allergies in urban than in rural environments, which has been associated with the differing microbial exposures between areas. However, potential similarities in the microbiota, that associate with environmental exposures, in allergic dogs and owners has not been investigated. We evaluated skin and gut microbiota, living environment, and lifestyle in 168 dog-owner pairs. Due to partly different manifestations of allergies between species, we focused on aeroallergen sensitized humans and dogs with owner-reported allergic symptoms. Our results agree with previous studies: dog-owner pairs suffered simultaneously from these allergic traits, higher risk associated with an urban environment, and the skin, but not gut, microbiota was partly shared by dog-owner pairs. We further discovered that urban environment homogenized both dog and human skin microbiota. Notably, certain bacterial taxa, which were associated with living environment and lifestyle, were also related with allergic traits, but these taxa differed between dogs and humans. Thus, we conclude that dogs and humans can be predisposed to allergy in response to same risk factors. However, as shared predisposing or protective bacterial taxa were not discovered, other factors than environmental microbial exposures can mediate the effect or furry dog and furless human skin select different taxa.

## Introduction

Pet dogs suffer from a variety of non-communicable diseases such as cancer, diabetes and allergic disorders^1^, which also burden human wellbeing in middle- and high-income countries^2^. To some extent, these diseases can manifest differently in dogs and humans, probably due to differences in immunological responses. For example, allergic dogs have skin and gut related symptoms while airway symptoms are rare, as opposed to humans.

An urban living environment predisposes both dogs and humans to allergic traits^3–7^. In humans, this association has been explained with the lack of allergy-protective factors in urban environments. For example, rare contacts with cattle and pets, small family size and westernized diets have been associated with a higher risk of allergic diseases^5^. Correspondingly, pet dogs have a reduced risk of allergies when exposed frequently to other animals as well as when living in a larger family^6^. Therefore, mammalian species may develop comparable disorders in response to similar factors. For this reason, pet dogs could serve as real-life models of diseases associated with environmental exposures.

Many of the above mentioned protective factors are assumed to increase the exposure to beneficial microbes, derived from natural and farming environments^8^. For example, farming-related house dust is associated with protection against asthma^9,10^. Moreover, dogs suffering from allergic symptoms have dissimilar skin microbiota than healthy individuals^7,11,12^, while humans suffering from asthma, allergic sensitization and atopic dermatitis tend to have dissimilar skin and/or gut microbiota structure compared to healthy individuals^13,14^.

These previous findings have primed our hypothesis that the effect of living environment and lifestyle in human and dog allergies can be the same. In support of this hypothesis, we have previously discovered in a large survey that cohabiting dogs and their owners tend to suffer from allergic traits or be healthy concurrently^6^. However, similarities in allergy risk have not been studied in detail. Especially, the role of microbiota as a common factor in the risk of allergic traits has not been addressed, although healthy dogs and their owners have been shown to partly share their skin microbiota^15^.

We have previously shown interlinkages between living environment, skin microbiota and allergic disorders separately in dogs and humans^7,16^. Here, we aim to investigate these interlinkages in 168 pairs of dogs and their owners to understand whether the effect of shared living environment, lifestyle and microbial exposures on risk of allergic traits is similar in cohabiting dogs and owners.

## Materials and methods

### Sample collection

A detailed description about the selection of participants and the sample collection is provided in Lehtimäki et al*.*^7^. Shortly, we contacted breeders of Finnish Lapphunds and Labrador’s Retrievers, who had litters born during 2012 or 2013, using the Finnish National Kennel Club register. Through these breeders the new owners of these puppies where recruited. Consequently, our data includes mother dogs (n = 39) and their owners (i.e. breeders, n = 39), adult puppies (n = 130) and their owners (n = 128). Thus, our data includes 168 dog-owner pairs. The characteristics of study population is presented at supplementary Table S1.

We invited owners and their dogs to sample collection events, which were organized during the autumn of 2014, in the southern parts of Finland. The skin microbiota of dogs was sampled from the inner side of the front leg, at about carpus level, which is anatomically similar to the volar forearm, where owners were sampled. Each skin sample was collected with a moisturized (sterile solution of 0.15 M NaCl and 0.1% Tween 20.) swab (Floqswabs, Copan flock technologies), by swabbing a small area several times up to down and from left to right, constantly rotating the swab. Whole blood was also collected from the forearm of both dog and owner into a glass serum tube, centrifuged, divided into 1 ml aliquots of sera and frozen in – 80 °C. Owners filled in questionnaires considering lifestyle, environment and possible allergic symptoms of their dogs^7^ and themselves (form S1). Allergic symptoms in dogs were defined using a large, validated questionnaire developed by veterinary dermatologists^7,17^ while in humans the definition of allergic symptoms was based on validated ISAAC^18^ questionnaire.

Owners collected fecal samples from their dogs and themselves in November 2014. They used a tube with spatula (Sarstedt #80.623.022) to sample a spoonful from the central part of the feces and placed the samples in their household freezer (− 18 °C) within an hour after sampling. Later, the samples were carried on dry ice from the owners’ residence to the lab. Unfortunately, we were unable to collect gut microbiota samples from 11 dogs and from 6 owners. Part of the owners (n_dogs_ = 96) also agreed to follow their dog’s exercise during a 1-week period, using a passive tracker (iTrail, SleuthGear). The exercise follow-up took place in spring and summer 2015.

### Sample processing

DNA from the skin microbiota samples was extracted and used as a template for amplification and subsequent sequencing of the V1–V3 16S rRNA gene region with Illumina MiSeq as previously described^19^. Gut microbiota samples were treated similarly, except that the DNA extraction was done with QIAamp DNA Stool Mini Kit (Qiagen) without modifications to the original protocol.

In humans, the common sensitization-causing allergens in Finnish adults were tested (birch, timothy, dog, cat and *Dermatophagoides pteronyssinus*) by The Skin and Allergy Hospital (Helsinki University Central Hospital) using the allergen specific Phadiatop test (ThermoFisher Scientific). Humans were defined as sensitized to aeroallergens if the sum of specific IgE for Timothy, Birch, Cat, Dog and *Dermatophagoides pteronyssinus* was > 0.99 kU/l. In dogs, sera samples were analyzed for several environmental allergens including different plants, molds, insects and house dust mite (listed in supplementary Table S2) specific Immunoglobulin Es (IgEs) (Avacta Animal Health, United Kindom). However, allergy in dogs was based on the severity of owner-reported symptoms, as reported previously^7^. Shortly, IgE values in dogs do not correspond well their symptoms, which is in agreement with previous reports^20,21^, and symptoms are regarded more reliable measure of allergy^7^.

### Quality control of the OTU data

The 16S rRNA gene sequences trimmed and further processed to cluster and annotate OTUS as previously described^7^. We excluded all non-bacterial sequences i.e. chloroplasts, mitochondria, and unknown at domain level as well as very rare (i.e. less than 10 sequences in the data) OTUs. Control samples from PCR and DNA extraction kits were used in the definition of potential contaminant OTUs. We also had blank controls collected from each sampling location, which were not used in the contaminant definition. Contaminants from PCR samples did not seem to cause problems in the data, but some OTUs originating from the DNA extraction kits were removed from skin and gut samples. The criteria to remove these contaminant OTUs were: (1) The OTU represents a suspicious group i.e. a known kit contaminant or it represents a microbial taxa that is unlikely to be present in skin or gut, or/and (2) the OTU seemed to cause patterns to the data i.e. its proportional influence was larger in samples containing smaller number of sequences (Table S3). Finally, we excluded all samples, which had less than 8000 sequences (n = 8) as we thought this was a reasonable limit, given that the largest sequence number in the control samples (blank control) was 7700.

As we wanted to control the effect of varied library sizes, we normalized the sample sizes. As the idea in this study was to compare two different species and two different body sites, we decided to normalize all these samples together. We used a cumulative sum square normalization provided in the metagenomeseq-package^22^, as this procedure reduced random noise but retained natural differences. The raw sequence data is provided in the National Center for Biotechnology Information (accession no. for dog skin: PRJNA434794, dog gut: PRJNA476220, human skin: PRJNA668051, and human gut: PRJNA668266). The metadata is available from the corresponding author at a reasonable request.

### Downstream statistical analysis

We simplified the information about the land cover in living and exercise environments with Principal Component Analysis (PCA) as previously^4,7,16^, and the lifestyle as well as the owner-reported allergic symptoms of dogs with PCoA using Gower’s distance. With this simplification approach, we ensured that we got most of the large variation to our use in objective yet easy-to-use form. The first axis in each analysis was extracted for the following analyses. The positive values along the land-use axes indicated a smaller proportion of built environment and an increased proportion of forested land-uses i.e. more rural land-use. Lifestyle was also defined as a gradient from urban (negative values) to rural (positive values) lifestyle. Features like small family size, no children in family, no other animal contacts or pets, living in high-rise apartment buildings and having many dog-related hobbies such as agility etc. correlated with an urban lifestyle while opposite features described the rural lifestyle.

All simplified variables were further categorized. Both the categorical land cover and lifestyle variables were divided into “rural” and “urban” by cutting the continuous variable into two similar sized parts. Dog-owner pairs had differing combinations of rural and urban environment and lifestyle i.e. 35% had both rural environment and lifestyle, 10% had rural environment but urban lifestyle, 20% had urban environment but rural lifestyle, and 35% had both urban environment and lifestyle.

As other complex information, also owner-reported symptoms of dogs were simplified with PCoA using Gower’s distance. The extracted first axis seemed to have a natural cut-off point (0.5), i.e. data had two peaks with a clear break (0.5) between those. It was used to divide the dogs into “allergic” and “healthy” as previously^7^. Humans were defined sensitized if their summed aeroallergen IgEs were at least 1 kU/l as previously^4^, and otherwise “healthy”. The relation between these categories and land-use and lifestyle variables were studied with general linear models, assuming binomial error variance (function glm in R). Due to significant correlation (collinearity) between living environment and lifestyle both in dogs (r = 0.48, p < 0.001) and owners (r = 0.49, p < 0.001), lifestyle was excluded from adjusted analyses.

We used the phyloseq package^23^ available for R to ease the concurrent handling of the OTU-table, the taxonomic information and the meta-data. We studied the basic features of the microbiota, i.e. the Shannon diversity and the dissimilarity between samples with the functions diversity and divergence provided in the vegan and microbiome R-packages, respectively^24,25^. In all dissimilarity-based analyses our response variable is Bray–Curtis dissimilarity matrix defined with vegdist function, provided in the vegan package. These analyses included unconstrained ordination with PCoA (function cmdscale) and constrained ordination with db-RDA (function capscale). Also, Permutational Multivariate Analysis of Variance Using Distance Matrices (function adonis) was used to uncover the effect of explanatory variables on dissimilarity matrices.

The machine learning method Random Forest regression (function randomForest in randomForest package^26^) was used to predict the extracted land cover and lifestyle axes from the microbiotas for getting an idea of how much these variables associate with microbiota structure. The Bayesian source tracking method developed in SourceTracker^27^ was used to study whether different microbiotas share microbes or can originate from each other. SourceTracker identifies the proportions of each source microbiota, including an unknown source, for each sink microbiota by comparing community structures in studied microbiotas. Finally, we searched the OTUs with dissimilar abundancies between allergic and healthy individuals as well as between rural and urban living environment or lifestyle with Zero-inflated Log-Normal mixture model provided in the metagenomeseq package^28^ (function fitFeatureModel). This method is specially developed for sparse microbiota data, and hence controls well potential false positives. Values below p = 0.05 were considered statistically significant. All statistical analyses were done in R version 3.5.2^29^.

### Ethics consideration

The collection of canine samples was ethically approved by the Animal Ethics Committee of the State Provincial Office of Southern Finland, Hämeenlinna, Finland (ESAVI/6054/04.10.03/2012). The dog owners’ sampling was approved by the Coordinating Ethics Committee, Helsinki and Uusimaa Hospital District (188/13/03/00/14). Sample collection and all subsequent experimental procedures were conducted in accordance with relevant guidelines and regulations. Before the sampling, we asked the owner of each dog to provide a signed informed consent, separately for their dog and themselves.

## Results

### Shared living environment and lifestyle associate with simultaneous allergic traits in dog-owner pairs

Of dog owners, 31% were sensitized to aeroallergens (mean sum of specific IgEs = 15.30 kU/l, range = [1.02, 78.94]). Interestingly, ca. 10% of owners were sensitized to dog, (i.e., specific IgE_dog_ > 0.35 kU/l), while none of the dogs were sensitized to humans. Aeroallergen sensitization in humans was significantly associated with self-reported symptoms of asthma (Fisher’s exact test, p = 0.04), eczema (p = 0.02), atopic dermatitis (p = 0.04), and wheeze (p = 0.003), while rhinitis showed borderline association (p = 0.07).

About 20% of dogs suffered from owner-reported allergic symptoms. In contrast to humans, IgE values were not a robust measurement of allergy, as sensitization was not associated with the severity of owner-reported symptoms (correlation = 0.06, p = 0.46).

**Figure 1:**
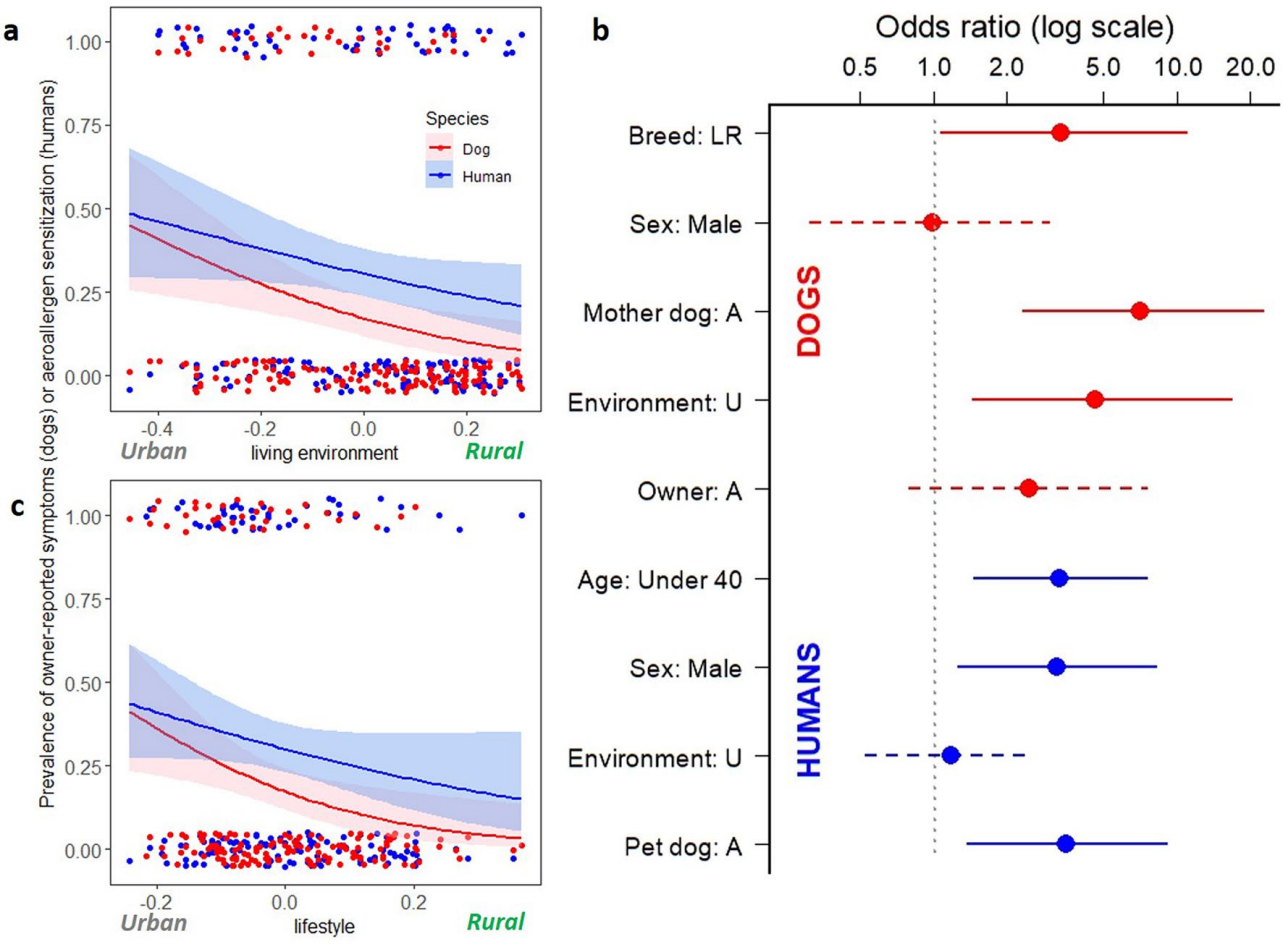
The association between living environment and lifestyle with allergic traits in dogs and humans. In dogs, both (**a**) living environment and (**c**) lifestyle associate with the prevalence of owner-reported allergic symptoms (logistic regression, p = 0.003 and p = 0.007, respectively) while in humans living environment and lifestyle showed borderline association with aeroallergen sensitization (p = 0.054 and p = 0.093, respectively). (**b**) Shows adjusted odds ratios from logistic regression separately for dogs and humans. Each explanatory variable is adjusted for other variables mentioned in the figure, separately for dogs and humans. Bars indicate the confidence intervals. Dashed bars indicate insignificant association between exposure and outcome in adjusted analysis. *Breed: LR* = breed of dog is Labrador’s Retriever, *Sex: Male* = Individual is male, *Mother dog: A* = dog’s mother is allergic, *Environment: U* = living environment of individual is urban, *Owner: A* = owner of a dog is sensitized, *Age: Under 40* = human is younger than 40 years, and *Pet dog: A* = pet dog suffers from owner-reported allergy symptoms.

Based on logistic regression, dogs living in urban areas were more likely to suffer from owner-reported allergic symptoms than rural dogs (p = 0.003) while similar but borderline association was discovered for humans (p = 0.054; Fig. 1a). As a sensitivity analysis, we excluded owners who reported they have allergic skin symptoms and tested association between dog allergy and living environment (p = 0.042), which suggest that the judgement of symptomatic owners did not confound our results. Similarly, urban-type lifestyle increased the risk of allergy in dogs (p = 0.007) while in humans association was not statistically significant (p = 0.093; Fig. 1c).

In accordance to previous research^6^, dogs and their owners tended to be allergic or healthy concurrently. In other words, an allergic dog was more likely to have a sensitized owner than a healthy dog (logistic regression, p = 0.002). The same was true for humans (p = 0.002), a sensitized owner was more likely to have an allergic than a healthy dog. This pattern did not change after exclusion of owners sensitized to dogs. After adjustments for age, sex, and living environment this association remained significant for humans (Fig. 1b, p = 0.009). Age and sex also associated with the risk of aeroallergen sensitization in humans (p = 0.004 and p = 0.015, respectively).

After adjustments for breed, sex, allergy of mother dog, and living environment, having an sensitized owner showed only a trend for dogs (p = 0.11). In dogs, being a Labrador retriever (instead of Finnish Lapphund), having an allergic mother dog, and living in an urban environment significantly increased the risk of allergy in the adjusted analysis (p = 0.042, p < 0.001, p = 0.013, respectively; Fig. 1b). However, large confidence intervals, arising from the small number of allergic dogs, warrants cautiousness to these results.

**Figure 2:**
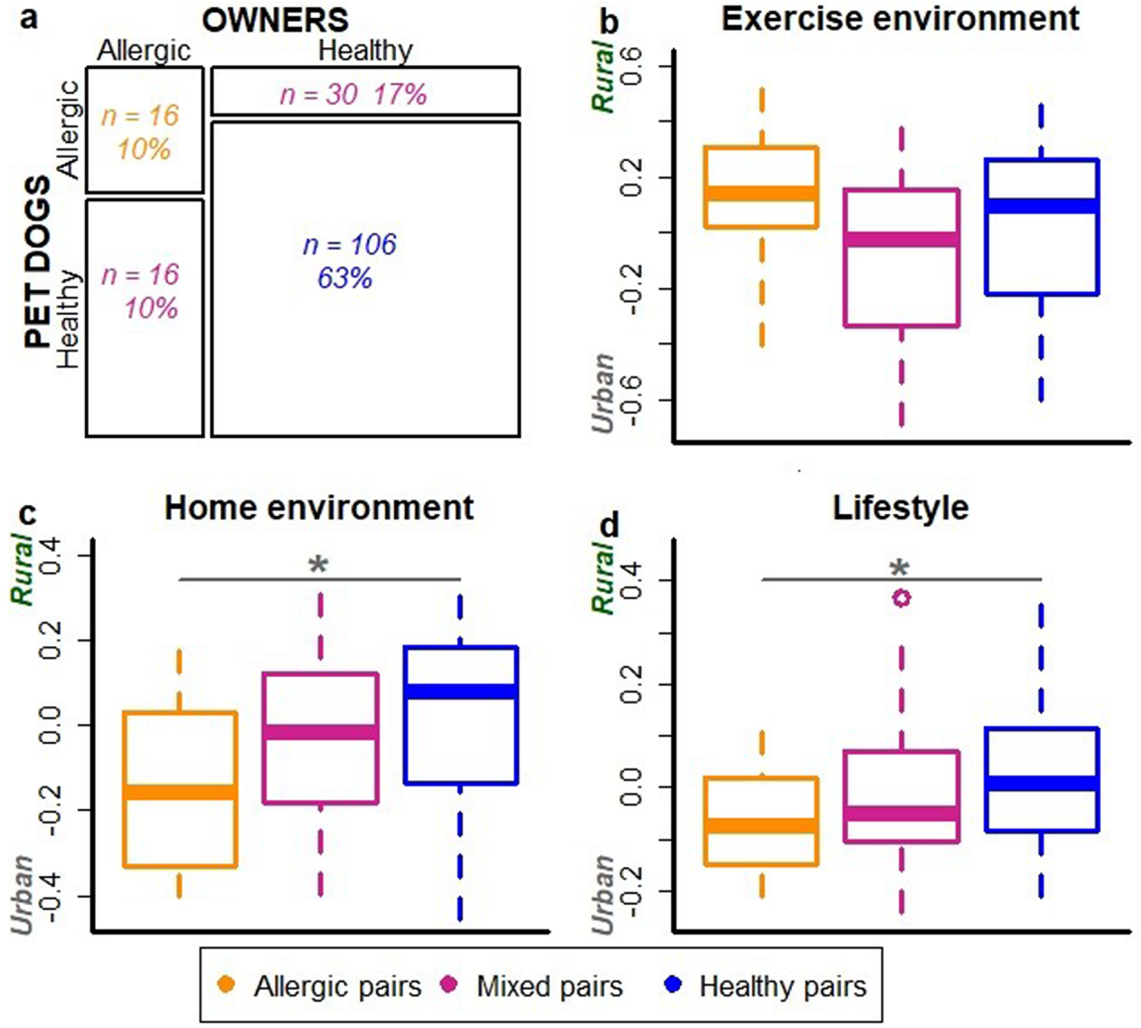
Dogs and their owners tend to be allergic or healthy concurrently. The proportion of allergic, healthy and mixed i.e. mixed dog-owner pairs in the data (**a**). The association of exercise environment (**b**), living environment (**c**) and lifestyle (**d**) with dog-owner groups. Decreasing values on y-axis indicate higher urbanization of exercise and living environment as well as more urban lifestyle. Living environment was significantly more urbanized in allergic than healthy dog owner pairs (ANOVA, p = 0.010). Additionally, lifestyle was more urban in allergic compared to healthy dog-owner pairs (p = 0.028).

We also divided dog-owner pairs into three groups: *allergic*, *healthy* and *mixed dog-owner pairs* (Fig. 2a). Mixed pairs included both healthy and allergic individual. These dog-owner groups associated with living environment and lifestyle; allergic dog-owner pairs were more likely to live in an urban environment and have urban-type lifestyle than healthy pairs (ANOVA, p = 0.010 and p = 0.028, respectively; Fig. 2c,d) while exercise environment did not show association (Fig. 2b).

### Dogs and their owners have partially shared microbiota on skin but not in gut

**Figure 3:**
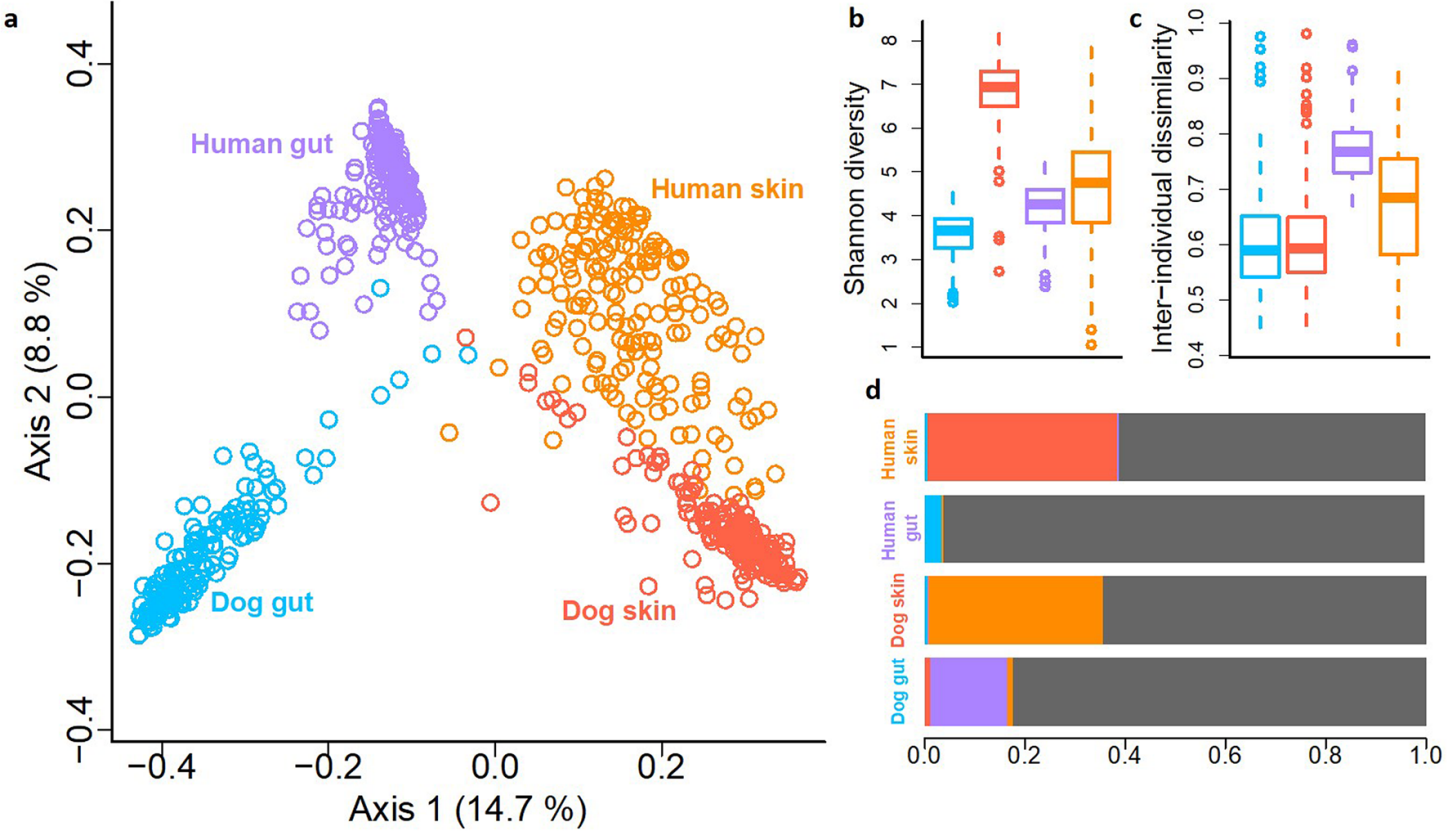
An overview of skin and gut microbiota differences between pet dogs and humans. The Principal coordinate analysis (**a**) shows separation between skin and gut microbiotas as well as between dogs and humans. Dog skin has clearly the highest Shannon diversity in comparison to other studied microbiotas (**b**). The inter-individual variation is higher between humans than dogs (**c**). The Bayesian source tracking analysis between studied microbiotas shows that skin microbiota of dogs and humans can originate from each other while gut microbiotas, especially in humans, are mostly independent (i.e. the proportion of unknown source, grey, was large) (**d**).

By analyzing skin and fecal samples from dog-owner pairs, we identified 52,589 operational taxonomic units (OTUs) from 43 million bacterial 16S rRNA gene sequences. Microbiotas tended to cluster primarily according to a body site, and secondarily according to species (Principal Coordinate Analysis, PCoA; Fig. 3a). The dog and human gut microbiotas were clearly separated, while the skin microbiotas of dogs and humans clustered closer to each other (Fig. 3a). *Fusobacteria* were highly abundant in the dog gut but largely absent in the human gut. *Proteobacteria* were more abundant on the dog skin than on the human skin, and rare phyla contributed more to dog than human skin microbiota (Fig. S1).

We studied associations between microbiotas utilizing Bayesian source tracking^27^. In agreement with previous analyses, the skin microbiota of human and dog were partly related to each other while gut microbiotas were largely independent (Fig. 3d). Indeed, the skin microbiota of dog-owner pairs resembled each other more than random dog–human pairs (ANOVA, p < 0.001, Fig. S2), while such an association was not found with the gut microbiota. Moreover, a larger proportion of OTUs were universally present in all studied skin samples (30 OTUs were present in 90% of all samples) than in all gut samples (9 OTUs were present in more than 60% of samples). For example, *Propionibacterium* was present in all but one skin sample.

The mean number of OTUs was clearly higher on the skin than in the gut in both dogs and humans (Table S4; p = 2.2e−16). Accordingly, the number of phyla, classes and families as well as proportion of unknown taxa were larger on the skin than in the gut of both hosts (Table S5). Among all habitats, the dog skin showed the highest richness (Table S4) and Shannon diversity (Fig. 3b; ANOVA, p < 0.0001 in each comparison). Interestingly, even though the dog skin was the most diverse habitat, the skin microbiota within dogs differed less between individuals than within humans (Fig. 3c; p = 1.53e−07), and this was also true for the gut microbiota (p < 2e−16).

### Structure of skin microbiota is associated with lifestyle and living environment in both species

Both living environment and lifestyle were more significantly associated with the skin than with the gut microbiota. Structure of skin microbiota differed between dogs and humans, rural and urban living environments and rural and urban lifestyle, while structure of gut microbiota differed only between dogs and humans. (PERMANOVA, Table S6).

Random Forest Regression (RFR) predicted twice as much variation in the living environment from dog skin compared to human skin microbiota (24.27% vs. 11.57% of out-of-bag (OOB) error variation, respectively). Only a small proportion could be predicted from the dog and human gut microbiotas (3.82% vs. 2.22% of OOB variation, respectively). Further, lifestyle was as predictable both from the dog and human skin microbiota (17.71% vs. 17.33% of OOB variation, respectively), while only the dog gut microbiota predicted lifestyle (15.66% vs. 1% of OOB variation).

Previously, we have reported homogenization of skin microbiota in urban dogs^7^. Also in dog owners, the skin microbiota was homogenized in urban environment and in response to urban lifestyle (Fig. S3a, p = 0.002, and Fig. S4a, p = 0.02, respectively). Interestingly, the opposite was true for their gut microbiota (Fig. S3b, p = 0.01).

We investigated whether the living environment and lifestyle are associated with the proportion of shared OTUs between dogs and humans. According to Source Tracking analysis, lifestyle seemed to explain the sharing of microbes, while living environment did not show associations. The urban lifestyle increased the proportion of human skin bacteria in the dog skin microbiota (p = 0.044; Fig. S5a). Moreover, rural lifestyle increased the proportion of the dog gut bacteria in the human gut microbiota (p = 0.028; Fig. S5b) as well as the proportion of human gut bacteria in the dog gut microbiota (p = 0.009; Fig. S5b).

### Modest similarities in the environment and lifestyle related microbiota in allergic dogs and sensitized humans

**Figure 4:**
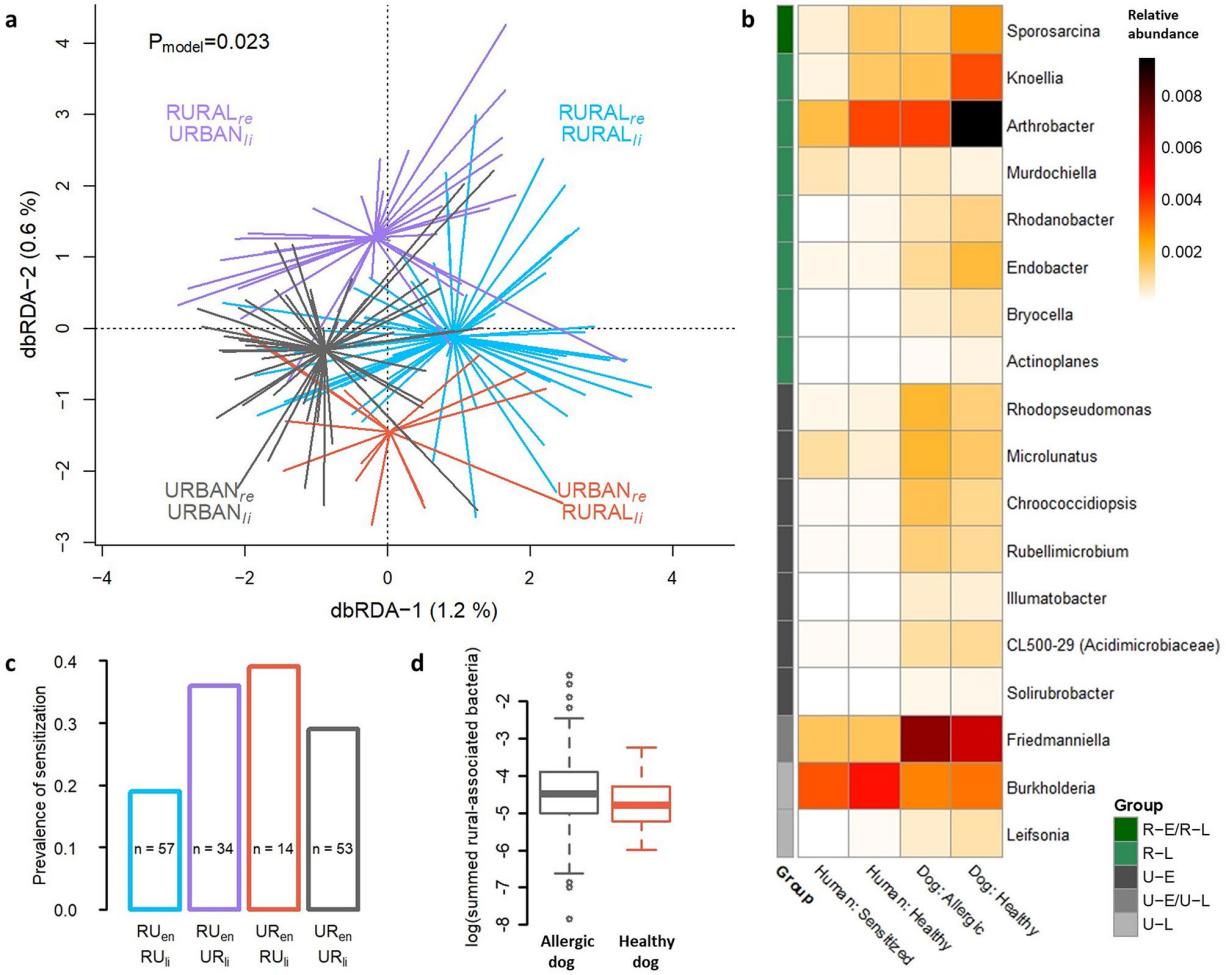
The living environment and lifestyle related skin microbiota in allergic and healthy individuals. Living environment (rural/urban) and lifestyle (rural-type/urban-type) constrained the skin microbiota of dog owners (**a**) in Distance-Based Redundancy Analysis. The prevalence of aeroallergen sensitization in humans showed association with the type of lifestyle and living environment (**c**) as extremes (rural environment + rural lifestyle vs. urban environment + urban lifestyle) were significantly different (ANOVA, p = 0.022). Bacterial genera in skin that significantly differed between rural and urban environments as well as between rural and urban lifestyles, defined with Zero-inflated Log-Normal mixture model, are presented as a heatmap of relative abundancies (**b**). Some of these taxa differ between species as well as between allergic and healthy individuals. RUen = Rural living environment, RUli = Rural lifestyle, URen = Urban living environment, URli = Urban lifestyle, re = residential environment, li = lifestyle, R-E/R-L = Bacteria associated with rural environment and rural lifestyle, R-L = Rural lifestyle associated bacteria, U-E = Urban environment associated bacteria, U-E/U-L = Urban environment and urban lifestyle associated bacteria, and U-L = Urban lifestyle associated bacteria. The log-transformed, summed relative abundance of rural-associated bacterial genera presented in heatmap were marginally lower in allergic than healthy dogs (p = 0.059; **d**).

We have previously discovered that skin microbiota is clustered by living environment and lifestyle in pet dogs, and that these factors are also associated with the risk of allergy^7^. Here, we observed corresponding clustering of skin microbiota in dog owners (Fig. 4a) as well as increasing prevalence of aeroallergen sensitization with the urbanization of living environment and lifestyle (Fig. 4c).

We searched genera that were dissimilarly abundant between allergic and healthy individuals as well as between rural and urban living environment or lifestyle. We did not find differentially abundant genera between healthy and allergic individuals. However, we found genera differentially abundant between rural and urban living environment and lifestyle, some of which further showed differing relative abundances between healthy and allergic individuals (Fig. 4b). The summed relative abundance of all rural-associated genera found in the analysis (Fig. 4b) were lower in the allergic than in the healthy dogs, but association was borderline (ANOVA, p = 0.059; Fig. 4d). Key taxa associated with allergic traits were distinct between species. Urban-environment associated *Rhodopseudomonas* was enriched in allergic dogs (p = 0.007) and rural-lifestyle associated *Actinoplanes* was enriched in healthy dogs (p = 0.02). *Artrobacter*, which associated with rural lifestyle in our data (and with farming in a previous study^9^), was more common in healthy than allergic dogs, but this associations were of borderline significance (ANOVA, p = 0.069). *Sprorosarcina,* that was associated with rural environment and lifestyle, was more common in healthy than in sensitized humans (p = 0.034; Fig. 4b).

We also studied weather the sharing of microbes between dogs and owners relate to allergic traits, based on Bayesian Source Tracking analysis. These analyses produced interesting, but mostly non-significant findings probably due to insufficient sample size. For example, increasing proportion of dog skin related bacteria on human skin showed trend towards decreasing prevalence of aeroallergen sensitization (Logistic regression, p = 0.104, Figs. S6a and S7a). On the other hand, allergic dogs tended to have a higher proportion of the human skin related bacteria than healthy dogs (p = 0.13, Figs. S6b and S7a). Interestingly, even though the median proportion of microbes relating to human skin was very low in the human gut (0.37%), it was significantly higher in allergic (0.43%) than in healthy (0.36%) humans (p = 0.003, Fig. S7b).

## Discussion

Our results suggested that dogs and owners were more likely to suffer simultaneously from allergic traits in urban environment and when having a lifestyle that limits exposure to environmental microbiota. Moreover, the skin microbiota tended to be shaped by the living environment in both species. However, our data did not show any significant microbial signal shared between species that concurrently associated with allergy in dogs and owners. For example, allergy-associated environmental bacterial taxa were not the same between humans and dogs. This is not unexpected, given the contrasting differences between human and dog skin as a habitat for microbiota as well as well-documented differences in immune tolerance. This neither exclude the potential for importance of environmental microbial exposures in the risk of allergies. For example, even though the environmental bacterial taxa associated to allergy were different between species, those can still have similar effects on immune function. However, this hypothesis requires further studies.

Indeed, our findings suggest the potential importance of microbial exposures in the risk of allergy both in dogs and humans. We draw this conclusion from our findings showing; (1) an association between skin microbiota and living environment as well as lifestyle, (2) an association between allergic traits and living environment as well as lifestyle, (3) homogenization of the skin microbiota in both species in urban environment, and (4) similar clustering of skin microbiota as well as the increase in the risk of allergy according to living environment and lifestyle in both species (Fig. 4a,c in owners and corresponding findings for dogs shown previously^7^). However, how significant these phenomena are for the *shared* risk of allergy in dogs and owners remains to be determined. A partly shared skin microbiota in dogs and their owners, including bacterial taxa which were enriched in both species in similar environments or with similar lifestyle, indicate that shared microbial exposures might have a role. Yet, also other predisposing or protective factors than microbial exposures can be important in the simultaneous allergic traits in dogs and humans. Rural and urban living environments and lifestyle differ in many ways, which our study did not address including, e.g. air pollution.

We argue that skin microbiota is under the influence of environmental microbial exposure^30^, more clearly in dogs than in humans, as indicated by the predictability of the living environment based on skin microbial structure. We found that dog skin microbiota is immensely diverse, supporting previous findings that taking a dog into the family results in a rapid microbial enrichment of house dust^31^. Furthermore, our data suggests that dog skin microbes might be enriched in the skin of healthy humans. Although this finding requires further support, it is in agreement with previous findings demonstrating mice elevated immune tolerance upon exposure to dog-associated house dust, mediated via modifications in their gut microbiota^32^. However, we propose that rather than resident dog skin microbes, the environmental microbes carried by dog skin/fur, have stronger immuno-regulatory potential. Fittingly, microbes on doormats are not related to dog ownership but rather to the type of living environment^33^, indicating that whether the dog carries protective or predisposing microbes indoors might depend on the environment. Interestingly, allergic dogs had more human skin related microbes on their skin. This could indicate decreased exposure to environmental microbes as enrichment of human skin microbes has been associated with indoor environments as well as to urban environments^34,35^.

In agreement with previous findings^15^, cohabiting dog-owner pairs did not share their gut microbes. The characteristic differences between human and dog gut microbiota are not unexpected, given their differences in physiology and diet. However, the importance of gut microbiota in allergic diseases should not be overlooked as previous research indicates that altered gut microbiota predisposes to allergic outcomes^36–39^. Further, information on factors that are known to shape gut microbiota structure, such as diet and medication, was limited in our study, leaving room for potential uncontrolled confounders.

Comparisons between human populations are often inflated by the large lifestyle variation, which makes it hard to estimate the source of differences. It is possible that the daily life of dogs has fewer changing factors than the daily life of humans. Humans often eat differently daily, may have a variety of medications, occupations and habits that increase the difference between individuals while average pet dogs eat more homogenous diets, more often stay inside the common home, and exercise in the close environment. Hence, even cross-sectional studies in dogs can provide interesting information about health-influencing features of environment. Therefore, comparison of pet dog populations could provide an interesting approach to understand, e.g., geographical health dissimilarities. The relevance of the pet dog as a model of human diseases has been discussed earlier^6,7,40^.

Besides our relatively small number of allergic individuals, leading to insufficient statistical power, our study is limited by the owner-reported allergy symptoms in the allergy definition of dogs. We utilized a validated questionnaire^7,17^ to diagnose allergy in dogs, as IgE in dogs does not seem to have the same role as IgE in humans^20,21^. Concurrent allergy may partly be explained by the increased sensitivity of allergic owners to notice and report allergy-related symptoms in their dogs. However, our sensitivity analysis did not support this possibility. Also, other potential biases such as participation bias may have influenced on our results. However, the participation rate was quite high (51% of invited dog owners^7^), which decreases possibility of such bias.

To our knowledge, concurrent allergies have not been reported in other cohabiting species pairs than dogs and their owners^6^. Also, obesity and increased long-term stress has also been reported to co-occur in dogs and owners^41,42^. Other non-communicable diseases such as diabetes and cancers are increasingly common both in dogs^43,44^ and humans. Interestingly, captive primates develop cardiovascular disorders in high rates^45^ for which environmental as well as diet related reasons have been suspected. Together with our findings, these examples highlight that the immune-related diseases can have common environmental origins, not only in humans, but also in other mammals.

## Supplementary Information

Supplementary Information.

## Data Availability

The raw sequence data is provided in the National Center for Biotechnology Information (accession no. for dog skin: PRJNA434794, dog gut: PRJNA476220, human skin: PRJNA668051, and human gut: PRJNA668266). The metadata is available from the corresponding author at a reasonable request.
