# Supplementary material for: Simultaneous allergic traits in dogs and their owners are associated with living environment, lifestyle and microbial exposures
Source: Sci Rep. 2020 Dec 15;10:21954. doi: 10.1038/s41598-020-79055-x (PMC7738549; doi:10.1038/s41598-020-79055-x)
Supplement: Supplementary file 1 — Supplementary Information. [file 41598_2020_79055_MOESM1_ESM.docx]

# SUPPLEMENTARY MATERIAL

# Simultaneous allergic traits in dogs and their owners are associated with living environment, lifestyle and microbial exposures

Jenni Lehtimäki^1,2^*, Hanna Sinkko^3^, Anna Hielm-Björkman^3^, Tiina Laatikainen^4,5,6^ Lasse Ruokolainen^7^, and Hannes Lohi^1,8,9^

^1^Research Programs Unit, Molecular Neurology, University of Helsinki, FI-00014 Helsinki, Finland

^2^Environmental Policy Centre, Finnish Environment Institute, FI-00790 Helsinki, Finland

^3^DogRisk and Helsinki One Health research groups, Department of Equine and Small Animal Medicine, Faculty of Veterinary Medicine, University of Helsinki, FI-00014 Helsinki, Finland

^4^National Institute for Health and Welfare, FI-00029 Helsinki, Finland

^5^Institute of Public Health and Clinical Nutrition, University of Eastern Finland, FI-70211 Kuopio, Finland

^6^Joint municipal authority for social and health care in North Karelia, FI-80210 Joensuu Finland

^7^Faculty of Biological and Environmental Sciences, University of Helsinki, FI-00014 Helsinki, Finland

^8^Department of Medical and Clinical Genetics; and Department of Veterinary Biosciences, University of Helsinki, FI-00014 Helsinki, Finland

^9^Folkhälsan Research Center, 00290, Helsinki, Finland

^*^Corresponding author, contact: [jenni.lehtimaki@environment.fi](mailto:jenni.lehtimaki@environment.fi)

## Supplementary figures

**Figure S1.** The relative abundance of the most abundant phyla in the data. The relative abundance is showed in each studied habitat i.e. human skin microbiota, human gut microbiota, dog skin microbiota and dog gut microbiota.

**Figure S2.** The dissimilarity between the skin microbiota in dog-owner pairs is smaller than in random pairs of dogs and humans (ANOVA, p <0.001). Dissimilarity is defined by Bray-Curtis metric.

**Figure S3.** The Bray-Curtis dissimilarity in the skin (a) and gut (b) microbiotas within rural and urban individuals. Boxplots represent median, interquartile range (the first and third quartiles), minimum and maximum values as well as outliers.

**Figure S4.** The Bray-Cutis dissimilarity in the skin (a) and gut (b) microbiotas within individuals with urban or rural lifestyle. Boxplots represent median, interquartile range (the first and third quartiles), minimum and maximum values as well as outliers.

**Figure S5.** The proportions of different microbiotas as sources of sink microbiota i.e. in the skin (a) and gut (b) microbiotas in relation to the lifestyle in dogs and humans defined with Bayesian Source Tracking analysis.


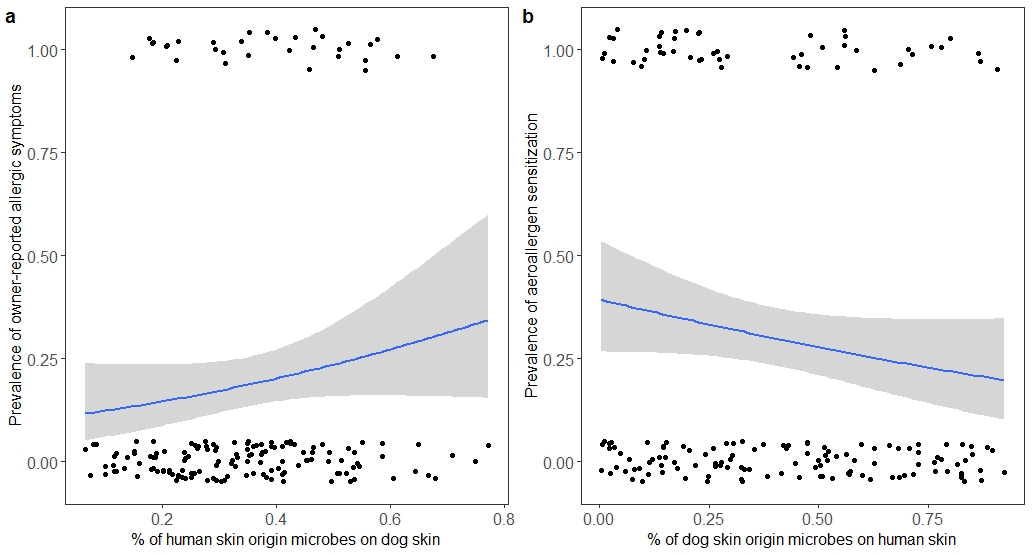


**Figure S6.** The relationship between the proportion of human-skin origin microbes on dog skin and the prevalence of owner-reported allergic symptoms in dogs (a). The relationship between the proportion of dog-skin origin microbes on human skin and the prevalence of aeroallergen sensitization (b).


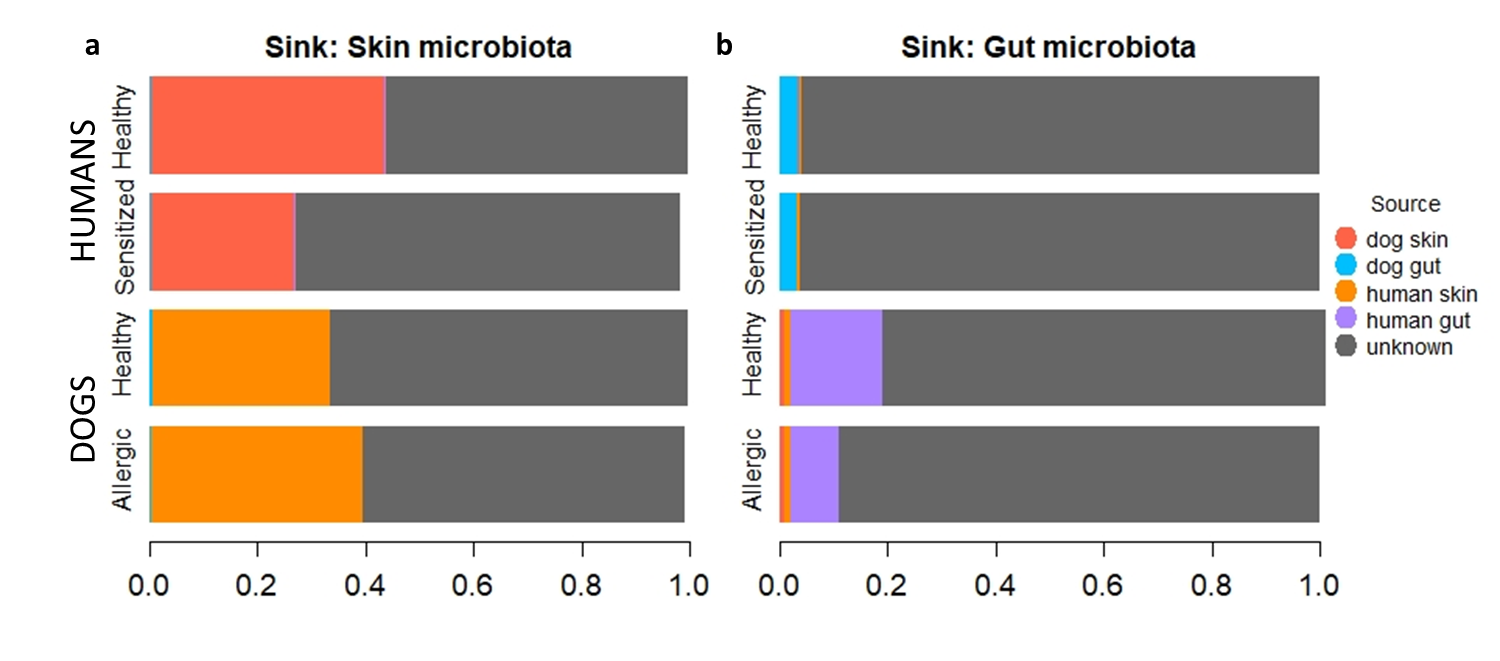


**Figure S7.** The proportions of different microbiotas as sources of sink microbiota i.e. in the skin (a) and gut (b) microbiotas in relation to allergy in dogs and humans defined with Bayesian Source Tracking analysis.

## Supplementary tables

**Table S1.** The characteristics of study population.

**Table S2.** List of specific immunoglobulin Es defined from the dogs. Note that values are expressed as Arbitrary Units (AUs) defined separately for each allergen by Avacta Animal Health (United Kingdom).

**Table S3.** Eight OTUs, defined from control samples, were removed from data as they were suspected to be contaminants. The criteria to define an OTU as a contaminant and remove it were: 1) an OTU represents suspicious group, i.e. it is a known contaminant from previous research or it belongs to microbial group uncommon in skin or gut such as microbe found from deep seas, 2) OTU seemed to cause pattern to the data, i.e. its proportional influence was larger in samples containing smaller number of sequences, and 3) OTU was only present in control samples. All OTUs that were removed based on the criteria 1 and 2 were defined from the blank controls made from the DNA extraction kit.

**Table S4.** Variation in the normalized sequence numbers and OTU numbers in different habitats i.e. the dog gut, dog skin, human gut and human skin. Sd = Standard deviation. Number of samples (n) is larger here than the actual number of study subjects due to few technical duplicate samples.

| **Species** | **Body site** |  | **Sequences** | **OTUs** |
| --- | --- | --- | --- | --- |
| DOG | Gut *n* = 159 | mean | 71685 | 405 |
|  |  | *sd* | *45015* | *327* |
|  | Skin *n* = 171 | mean | 73328 | 5546 |
|  |  | *sd* | *45480* | *2515* |
| HUMAN | Gut *n* = 170 | mean | 61035 | 669 |
|  |  | *sd* | *36089* | *221* |
|  | Skin *n* = 171 | mean | 50879 | 2035 |
|  |  | *sd* | *26213* | *1441* |

**Table S5.** Number of taxa in each habitat. *n different* indicates number of taxa that differ between humans and dogs. *n dominant* indicates the number of taxa that had more than 2 % relative abundance of the OTUs in given habitat.

|  | **Phyla** | **Class** | **Family** | **Unknown %** | **n dominant** |
| --- | --- | --- | --- | --- | --- |
| SKIN | 35 | 90 | 409 | 34 | 7 |
| *Human* | 34 | 82 | 374 | 31 | 5 |
| *Dog* | 35 | 90 | 405 | 35 | 1 |
| *n different* | *1* | *8* | *35* |  |  |
| GUT | 28 | 59 | 215 | 24 | 17 |
| *Human* | 22 | 43 | 160 | 23 | 6 |
| *Dog* | 25 | 54 | 192 | 23 | 10 |
| *n different* | *6* | *31* | *55* |  |  |

**Table S6.** Permutational Multivariate Analysis of Variance (PERMANOVA) utilizing Bray-Curtis sample-wise dissimilarities for skin and gut microbiota. Analysis was limited to individuals who had an exercise follow-up.

|  | **Explanatory variable** | **R2** | **p** |
| --- | --- | --- | --- |
| SKIN n = 160 | Species | 0.169 | **0.001** |
|  | Living environment | 0.009 | **0.029** |
|  | Lifestyle | 0.009 | **0.028** |
|  | Exercise environment | 0.005 | 0.298 |
| GUT n = 149 | Species | 0.19894 | **0.001** |
|  | Living environment | 0.007 | 0.136 |
|  | Lifestyle | 0.006 | 0.157 |
|  | Exercise environment | 0.005 | 0.458 |

## Supplementary forms

**Form S1.** Questionnaire for dog owners considering their own symptoms and background. Questionnaire for dog symptoms, lifestyle and living environment can be found from the supplementary material of previously published study^1^.

1. **Basics**
2. Date
3. ID
4. Birthday
5. Gender
6. Pet dog(s)

1 I have always had dog(s)

2 I have had dog(s) for years

3 I have never had a dog

**2a. Respiratory symptoms**

1. Has there **ever** been wheezing sound in your breath?

1 No

2 Yes

1. Has there been wheezing sound in your breath **during the last twelve months**?

1 No

2 Yes

1. Have you **ever** had asthma?

1 No

2 Yes, according my own judgement

3 Yes, diagnosed by a doctor

1. Have you take medicine for treatment of asthma **during the last twelve months**?

1 No

2 Yes

3 I don’t have a asthma

1. How many times **during the last 12 months** you had had a respiratory infection?

1 None

2 1

3 2-4

4 more than 4

**2b. Rhinitis symptoms**

All questions in this part concern the situation when you are NOT having flu or respiratory tract infection!

1. Have you **ever** had sneezing, nasal congestion or sniffles when NOT having a flu or

respiratory tract infection?

1 No

2 Yes

1. Have you had sneezing, nasal congestion or sniffles when NOT having a flu or respiratory tract infection **during the last twelve months**?

1 No

2 Yes

1. Have you had itchy or watery eyes in connection with these rhinitis symptoms **during the last twelve months**?
2. No
3. Yes
4. In which month(s) these rhinitis symptoms occurred **during the last twelve months** (you can choose many)?

1 January

2 February

3 March

4 April

5 May

6 June

7 July

8 August

9 September

10 October

11 November

12 December

1. When you had had hay fever (you can choose many)?

1 Less than a year old

2 1-10 years old

3 10-15 years old

4 more than 15 years old

5 I have never had hay fever

1. If you have had hay fever, was it diagnosed by a medical doctor?

1 No

2 Yes

3 I have never had hay fever

**2c. Eczema symptoms**

1. Have you **ever** had an itchy rash?

1 No

2 Yes

1. Have you had an itchy rash **during the last twelve months**?

1 No

1. Yes
2. Have you **ever** had itchy rash in some of the following sites*: in front of elbows, in back of knees, front side of ankles, buttocks, neck, or around of eyes or ears*?

1 No

2 Yes, constantly

3 Yes, but it disappears every now and then

1. Have you had a atopic dermatitis?

1 No

2 Yes, according my own judgement

3 Yes, diagnosed by a medical doctor

1. If you have had eczema symptoms during the last 12 months, which type of treatments or medicines you have utilized?

**2d. Symptoms of relatives**

1. Did your **mother** had following diseases?

**Yes, Yes, No According own diagnosed by I don’t know**

**judgement a doctor**

Allergic rhinitis □ □ □ □

Asthma □ □ □ □

Atopic dermatitis □ □ □ □

1. Did your **father** had following diseases?

**Yes, Yes, No According own diagnosed by I don’t know**

**judgement a doctor**

Allergic rhinitis □ □ □ □

Asthma □ □ □ □

Atopic dermatitis □ □ □ □

1. Did your **siblings** have following diseases?

I don’t have siblings □

**Yes, Yes, No According own diagnosed by I don’t know**

**judgement a doctor**

Allergic rhinitis □ □ □ □

Asthma □ □ □ □

Atopic dermatitis □ □ □ □

1. Do your **children** have following diseases?

I don’t have children □

**Yes, Yes, No According own diagnosed by I don’t know**

**judgement a doctor**

Allergic rhinitis □ □ □ □

Asthma □ □ □ □

Atopic dermatitis □ □ □ □

1. **Family and lifestyle during childhood**

***Note!*** *Following questions regard the time when* ***you were under 18 years old!***

1. I have _____ older siblings

and _____ younger siblings.

1. Which kind of education your mother and father have?

**mother father**

Elementary or primary school □ □

Middle or vocational school □ □

High school or college □ □

University degree □ □

1. Did anybody who spend time with you daily smoke?

1 No

2 Yes

1. Did you get exposed to cigarette smoke indoors?

1 No

2 Yes

1. Birth weight in grams
2. Were you breastfed?

1 No

2 Yes, about_____ months

1. **Current lifestyle**
2. Do you smoke cigarrettes?

1 Yes, I have smoked more than 5 years

2 Yes, I have smoked less than 5 years

3 I have never smoked

4 I have quitted

1. Have you had antibiotics during the last 12 months?

1 No

2 Yes, How many times? _______

1. Do you have following food allergies?

No Yes I don’t know

fish □ □ □

egg □ □ □

milk □ □ □

grains □ □ □

citrus fruits □ □ □

nuts □ □ □

apple □ □ □

carrot □ □ □

1. Do you have other allergies such as animal and medicine allergies?

_____________________________________________________________________

1. **Living environment during childhood**

***Note!*** *Following questions regard the time when* ***you were under 18 years old!***

1. Have you lived in the same apartment when under 18 years old?

1 Yes

2 No

**→ If you answered yes**:

address:

**→ If you answer no:**

address of apartment where you spend the longest time period during childhood:

I was living here when __-__ years old

address of apartment where you spend the second longest period during childhood:

I was living here when __-__ years old

1. Primary home located at

1 City center

2 Municipality center

3 Suburban

4 Conurbanitation

5 Sparsely populated area

1. Primary home was

1 in high-rise apartment building

2 rowhouse

3 town or twinhouse

4 farm

1. Primary home

1 had yard

2 had shared yard

3 had no yard

1. During childhood I was spending time outdoors outside of home yard

1 daily

2 almost daily

3 1-3 time a week

4 less than once a week

1. Did your family practice farming?

1 No

2 Yes, full-time

3 Yes, part-time

1. Did you have a dog?
2. No
3. Yes
4. If you answered yes, when did you have a dog?

**No Yes I don’t know**

0-5 years old □ □ □

5-10 years old □ □ □

10-18 years old □ □ □

1. Did you have a cat?

1 No

2 Yes

1. If you answered yes, when did you have a cat?

**No Yes I don’t know**

0-5 years old □ □ □

5-10 years old □ □ □

10-18 years old □ □ □

1. Did you have some other pet(s)?

1 No

2 Yes

1. If yes, which animal it was?
2. If you answered yes, when did you have these pet(s)?

**No Yes I don’t know**

0-5 years old □ □ □

5-10 years old □ □ □

10-18 years old □ □ □

1. Did you have a regular (at least weekly) contact with animals owned by others?
2. No
3. Yes
4. If yes, which animal this was?
5. Did you have a contact with farm animals cow, pig, horse, coat, sheep etc.) during childhood?

1 Daily

2 At least once a week

3 At least once a month

4 At least once a year

5 Rarely or never

1. **Current living environment**
2. Current address:
3. I have lived in current address for __ years and __ months
4. During my life, I have lived

1 Only countryside

2 Only in cities

3 Both countrysice and cities

1. **Contact with nature during childhood**

***Note!*** *Following questions regard the time when* ***you were under 18 years old!***

1. Did your family have rural second home (summer cottage)?
2. No
3. Yes
4. If yes, how many weeks on average you spend there yearly?
5. During childhood, how many weeks yearly did you spend on average at rural areas visiting relatives?
6. More than twice a year our family did following activities (you can choose many)?

1 picking mushrooms

2 picking berries

3 fishing

4 hiking

5 boat trip

6 hiking overnight

1. **Current contact with nature**
2. Choose

□ I visit yearly at rural second home

□ I don’t visit yearly at rural secnd home

1. How many weeks you spend at rural second home yearly on average?
2. How many weeks you spend at rural areas visiting relatives yearly on average?
3. More than twice a year our family did following activities (you can choose many)?

1 picking mushrooms

2 picking berries

3 fishing

4 hiking

5 boat trip

6 hiking overnight

1. Feedback:

## Supplementary references

1. Lehtimäki, J. *et al.* Skin microbiota and allergic symptoms associate with exposure to environmental microbes. *PNAS* **115**, 4897–4902 (2018).
